# Whole-genome discovery of pathogenic snRNA variants and efficient extended-exome screening

**DOI:** 10.1016/j.isci.2026.116814

**Published:** 2026-07-16

**Authors:** Yuka Nakano, Hisato Suzuki, Yukiko Kuroda, Hiroshi Yoshihashi, Nobuhiko Okamoto, Akane Kondo, Rika Kosaki, Kenichi Kashimada, Toshihide Kurihara, Meow-Keong Thong, Sok-Kun Tae, Mazlan Rifhan, Takashi Enokizono, Hiroshi Suzumura, Takeshi Yoshida, Shinji Kosugi, Seiji Mizuno, Mie Inaba, Natsuki Nakamura, Mayumi Matsufuji, Eri Ogawa, Hitomi Yagi, Mamiko Yamada, Emi Qian, Daisuke Nakato, Toshiki Takenouchi, Kenjiro Kosaki, Fuyuki Miya

**Affiliations:** 1 Center for Medical Genetics, Keio University School of Medicine, Tokyo, Japan; 2 Department of Clinical Medicine, Institute of Medicine, University of Tsukuba, Ibaraki, Japan; 3 Division of Medical Genetics, Kanagawa Children’s Medical Center, Yokohama, Japan; 4 Department of Clinical Genetics, Tokyo Metropolitan Children’s Medical Center, Fuchu, Tokyo, Japan; 5 Department of Medical Genetics, Osaka Women’s and Children’s Hospital, Osaka, Japan; 6 Perinatal Medical Center, Shikoku Medical Center for Children and Adults, National Hospital Organization, Kagawa, Japan; 7 Division of Medical Genetics, National Center for Child Health and Development, Tokyo, Japan; 8 Division of Endocrinology and Metabolism, National Center for Child Health and Development, Tokyo, Japan; 9 Department of Ophthalmology, Keio University School of Medicine, Tokyo, Japan; 10 Genetics Medicine Unit, Universiti Malaya Medical Centre, Kuala Lumpur, Malaysia; 11 Genetics and Metabolism Unit, Department of Paediatrics, Faculty of Medicine, Universiti Malaya, Kuala Lumpur, Malaysia; 12 M. Kandiah Faculty of Medicine and Health Sciences, Universiti Tunku Abdul Rahman, Bandar Sungai Long, Kajang, Selangor, Malaysia; 13 Department of Child Health, Institute of Medicine, University of Tsukuba, Ibaraki, Japan; 14 Department of Pediatrics, Dokkyo Medical University, Tochigi, Japan; 15 Department of Pediatrics, Kyoto University Graduate School of Medicine, Kyoto, Japan; 16 Department of Genomic Medicine, Graduate School of Medicine, Kyoto University, Kyoto, Japan; 17 Department of Pediatrics, Central Hospital, Aichi Developmental Disability Center, Kasugai, Japan; 18 Department of Pediatrics, Kagoshima City Hospital, Kagoshima, Japan; 19 Department of Pediatrics, Keio University School of Medicine, Tokyo, Japan; 20 Department of Pediatrics, Federation of National Public Service Personnel Mutual Aid Associations Tachikawa Hospital, Tokyo, Japan; 21 Department of Pediatric Neurology, Okayama University Graduate School of Medicine, Dentistry and Pharmaceutical Sciences, Okayama, Japan; 22 Department of Precision Cancer Medicine, Center for Innovative Cancer Treatment, Institute of Science Tokyo Hospital, Tokyo, Japan

**Keywords:** RNU4-2, RNU5B-1, RNU2-2, RNU4ATAC, snRNA, small nuclear non-coding RNA, whole-genome sequencing, extended whole-exome sequencing

## Abstract

Pathogenic variants in small nuclear RNA (snRNA) genes have recently emerged as a major cause of Mendelian disorders, particularly neurodevelopmental disorders, yet they remain difficult to detect in routine diagnostics because conventional whole-exome sequencing (WES) does not capture snRNA loci. Here, we reanalyzed whole-genome sequencing (WGS) data from 1,578 unsolved probands and identified pathogenic variants in multiple snRNA genes, including *RNU4-2*, *RNU2-2*, *RNU5B-1*, and *RNU4ATAC*, accounting for 1.2% (19 patients) of previously unsolved cases. We then developed an snRNA-extended WES approach by incorporating capture probes targeting 50 snRNA genes into a standard exome design. Benchmarking demonstrated robust, uniform coverage across all targeted snRNA loci without increasing sequencing depth. Applying this approach to patient samples reliably detected disease-causing snRNA variants previously identified by WGS. Our results establish snRNA-extended WES as a cost-effective and scalable strategy to improve diagnostic yield and bridge the gap between recent gene discoveries and clinical genomic practice.

## Highlights

• WGS identified pathogenic variants in *RNU4-2*, *RNU2-2*, *RNU5B-1*, and *RNU4ATAC*
• snRNA variants explained 1.2% of previously genetically unsolved cases
• snRNA-extended WES captured 50 snRNA genes without increasing sequencing depth
• snRNA-extended WES is a cost-effective, scalable route to improve diagnostic yield

## Graphical abstract

**Figure:**
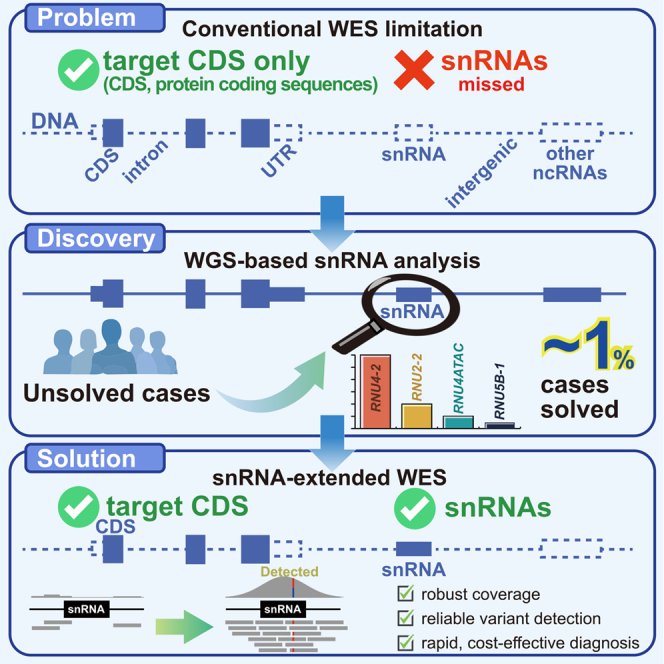

## Teaser

Human genetics; Precision medicine; Genomics

## Introduction

Mendelian diseases have traditionally been attributed to nonsynonymous variants in protein-coding genes or to structural alterations affecting genes of interest. However, a growing body of evidence has highlighted the role of small nuclear RNAs (snRNAs), a class of non-coding RNA, as causal factors in monogenic disorders. snRNAs are short non-coding RNAs of approximately 150 nucleotides that function primarily as essential components of the spliceosome and that play a critical role in pre-mRNA splicing.^1^ Over the past two years, this field has expanded rapidly, with pathogenic or likely pathogenic variants reported in nine additional snRNA genes, including *RNU4-2*,^2^^,^^3^ *RNU6ATAC*,^4^^,^^5^ four identical paralogs of *RNU6* (*RNU6-1*, *RNU6-2*, *RNU6-8*, and *RNU6-9*),^6^ *RNU2-2*,^7^^,^^8^ *RNU5B-1*,^8^^,^^9^ and *RNU5A-1*.^9^ Neurodevelopmental disorders (NDDs) caused by *RNU4-2* variants, also known as ReNU syndrome, has been estimated to account for approximately 0.4% of currently undiagnosed cases of NDDs worldwide,^3^ representing a larger contribution than that of any other single gene associated with intellectual disability reported to date. Although *RNU2-2* variants are less frequent, their prevalence has been estimated to be approximately 20% of that observed for *RNU4-2*,^7^ underscoring the substantial impact of these recent discoveries. In addition, autosomal recessive disorders associated with *RNU4-2*^10^^,^^11^ and *RNU2-2*^12^^,^^13^^,^^14^ have recently been reported. Notably, recessive *RNU2-2* syndrome has been proposed as one of the most prevalent recessive NDDs identified to date,^12^ further emphasizing the critical role of snRNA genes in human development. Moreover, independent studies have highlighted the functional breadth of snRNA genes by identifying retinitis pigmentosa-causing variants in *RNU4-2* and in four paralogs of *RNU6*.^6^ Another recent study^5^ implicated the minor spliceosome in immune function by identifying pathogenic variants in *RNU4ATAC* and *RNU6ATAC* underlying neonatal diabetes. Together with previously established disease-associated snRNAs, including *RNU7-1*,^15^ *RNU12*,^16^^,^^17^ and *RNU4ATAC*,^18^^,^^19^^,^^20^^,^^21^ these findings firmly establish snRNA genes as an emerging and important class of Mendelian disease genes.

Despite the growing recognition of pathogenic snRNA variants, accessible and rapid assays for their detection remain limited. Because commercial whole-exome sequencing (WES) capture probes are typically designed to target only the coding sequence (CDS) regions of protein-coding genes, current WES approaches cannot reliably detect variants in snRNAs.^3^ Consequently, identification of snRNA variants has thus far relied on labor-intensive and costly methods, such as gene-specific PCR amplification followed by Sanger sequencing or whole-genome sequencing (WGS) with downstream variant calling analysis.

We have recently developed a cost-effective strategy named “extended WES,” which expands WES target regions to include intronic regions of disease-associated genes as well as loci implicated in repeat expansion disorders, thereby improving the sensitivity of structural variant (SV) detection.^22^ This simultaneously expands the target design of WES while maintaining its efficiency, thereby improving its diagnostic yield.

In this study, we first reanalyzed WGS data from our cohort to identify pathogenic variants in snRNA genes. We then assessed whether incorporation of snRNA genes into our extended WES design enables rapid and cost-effective screening for disease-causing snRNA variants.

## Results

### Retrospective analysis of WGS data

**Figure 1:**
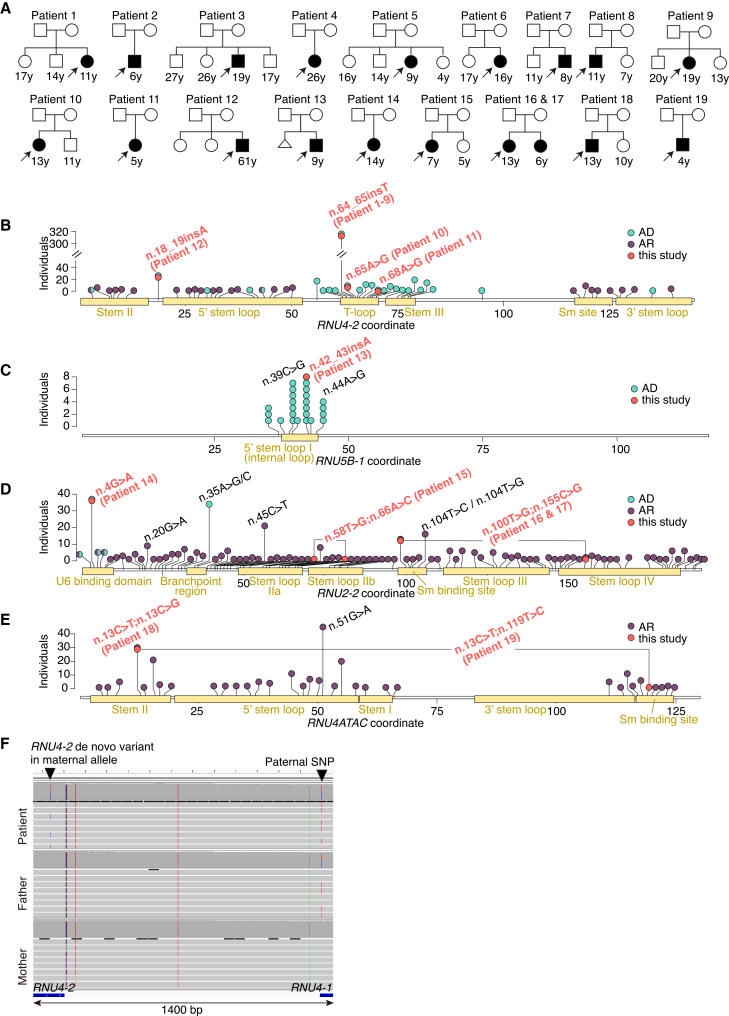
Identified pathogenic and likely pathogenic variants (A) Pedigrees of identified probands. (B–E) Lollipop plots showing the positions and frequencies of previously reported pathogenic variants together with variants identified in this study in *RNU4-2* (B), *RNU5B-1* (C), *RNU2-2* (D), and *RNU4ATAC* (E). Previously published studies reporting the variants used for figure generation are summarized in Table S1. (F) Representative example of parental origin analysis of a *de novo* variant using nanopore long-read sequencing. An integrative genomics viewer (IGV) screen is shown in which colored bases indicate mismatches relative to the reference sequences and each gray horizontal bar represents an individual sequencing read. The *de novo RNU4-2* variant is observed exclusively in reads lacking the paternal variant, indicating that the variant arose on the maternal allele. SNP, single nucleotide polymorphism.

We reanalyzed WGS data from 1,578 probands without previously identified disease-causing variants, including those with NDD. This reanalysis identified pathogenic variants in *RNU4-2* in 12 patients, *RNU5B-1* in one patient, *RNU2-2* in four patients, and *RNU4ATAC* in two patients (Figure 1A; Table 1; Figure S1). Among these variants, nine had been previously reported, whereas four were newly identified in this study (Figures 1B–1E; Tables 1 and S1). Overall, screening of snRNA genes revealed pathogenic variants in 1.20% of previously genetically unsolved cases (19 of 1,578 probands).

**Table 1:** Identified variants

Patient	Patient 1-9	Patient 10	Patient 11	Patient 12	Patient 13	Patient 14	Patient 15	Patient 16 and 17	Patient 18	Patient 19
Gene	*RNU4-2*	*RNU4-2*	*RNU4-2*	*RNU4-2*	*RNU5B-1*	*RNU2-2*	*RNU2-2*	*RNU2-2*	*RNU4ATAC*	*RNU4ATAC*
Genomic coordinate (hg38)	chr12: 120291838_ 120291839	chr12: 120291839	chr12: 120291836	chr12: 120291885_ 120291886	chr15: 65304718_ 65304719	chr11: 62841806	chr11: 62841752; 62841744	chr11: 62841710; 62841655	chr2: 121530892	chr2: 121530892; 121530998
Transcript ID	NR_003137.3	NR_003137.3	NR_003137.3	NR_003137.3	NR_002757.3	NR_199791.1	NR_199791.1	NR_199791.1	NR_023343.3	NR_023343.3
HGVS nomenclature	n.64_65insT	n.65A>G	n.68A>G	n.18_19insA	n.42_43insA	n.4G>A	n.58T>G; n.66A>C	n.100T>G; n.155C>G	n.13C>G; n.13C>T	n.13C>T; n.119T>C
Pathogenic reporting status	known	known	novel	known	known	known	novel; novel	known; known	known; known	known; novel
Zygosity	heterozygous	heterozygous	heterozygous	heterozygous	heterozygous	heterozygous	compound heterozygous	compound heterozygous	compound heterozygous	compound heterozygous
Mode of inheritance	*de novo* (AD)	*de novo* (AD)	*de novo* (AD)	AD (suspected)	*de novo* (AD)	*de novo* (AD)	AR	AR	AR	AR
gnomAD^35^ AF (v.4.1)	0	0	0	0	0	0	6.57e−6; 0	2.63e−5; 2.63e−5	0; 0	0; 0
jMorp^36^ AF (61K WGS)	0	0	0	0	0	0	0; 8.00e−6	0; 1.60e−5	8.00e−6; 5.86e−4	5.86e−4; 8.00e−6
Variant-bearing allele	maternal (all 9 cases)	maternal	maternal	maternal (suspected)	maternal	maternal	paternal; maternal	paternal; maternal	maternal/paternal	maternal; paternal

AD, autosomal dominant; AR, autosomal recessive; AF, allele frequency; jMorp, Japanese multi-omics reference panel database (https://jmorp.megabank.tohoku.ac.jp). Italicized entries indicate gene symbols and the Latin term '*de novo,*' which are conventionally italicized.

Among the patients harboring *RNU4-2* variants, nine carried the highly recurrent n.64_65insT (NR_003137.3) variant, one carried the rare n.65A>G variant, one carried the novel n.68A>G variant, and one carried the n.18_19insA variant (Figure 1B; Tables 1 and 2). All variants except n.18_19insA were located within the T-loop region, a known mutational hotspot also referred to as a critical region.^3^ The associated phenotypes were consistent with previous reports and included psychomotor developmental delay, hypoplasia of the corpus callosum, characteristic facial features, and epilepsy (Table 2; see also Document S1). In contrast, n.18_19insA was located within the region between stem II and the 5′ stem loop and was associated with a markedly distinct phenotype. This variant has recently been reported to cause retinitis pigmentosa with familial inheritance.^6^ The patient described here showed no sign of NDD but presented with Brugada syndrome, a rare inherited cardiac disorder, together with retinitis pigmentosa. Prior to the present reanalysis, *SCN5A* (NM_000335.5):c.239T>A,p.(Leu80Gln) had been identified as the causative variant for Brugada syndrome. However, no causative variant for retinitis pigmentosa had been identified, and the case had remained genetically unsolved with respect to retinitis pigmentosa. Subsequently, this reanalysis identified *RNU4-2*:n.18_19insA as the causative variant for retinitis pigmentosa, resulting in a diagnosis of dual etiology (Document S1).

**Table 2:** Clinical features of patients identified in this study stratified by snRNA gene

System	Feature	*RNU4-2*^a^	*RNU5B-1*	*RNU2-2*	*RNU4ATAC*
Prenatal findings	abnormal cerebral findings	63.6% (7/11)	0.0% (0/1)	0.0% (0/4)	50.0% (1/2)
	IUGR	36.4% (4/11)	0.0% (0/1)	0.0% (0/4)	100.0% (2/2)
Neonatal findings	neonatal feeding problems	63.6% (7/11)	100.0% (1/1)	75.0% (3/4)	N/A
	neonatal hypotonia	60.0% (6/10)	100.0% (1/1)	50.0% (2/4)	N/A
Growth	microcephaly	72.7% (8/11)	0.0% (0/1)	25.0% (1/4)	0.0% (0/2)
	short stature	63.6% (7/11)	0.0% (0/1)	0.0% (0/4)	100.0% (2/2)
Neurodevelopmental	ambulation	not achieved: 45.5% (5/11)	not achieved: 0.0% (0/1)	not achieved: 25.0% (1/4)	not achieved: 0.0% (0/2)
		delayed: 54.5% (6/11)	delayed: 100.0% (1/1)	delayed: 75.0% (3/4)	delayed: 50.0% (1/2)
		normal development: 0.0% (0/11)	normal development: 0.0% (0/1)	normal development: 0.0% (0/4)	normal development: 50.0% (1/2)
	autistic spectrum disorder	60.0% (6/10)	0.0% (0/1)	25.0% (1/4)	0.0% (0/2)
	brain MRI abnormalities	100.0% (10/10)	100.0% (1/1)	0.0% (0/2)	100.0% (2/2)
	developmental delays	severe: 90.9% (10/11)	severe: 100.0% (1/1)	severe: 75.0% (3/4)	severe: 0.0% (0/2)
		moderate: 9.1% (1/11)	moderate: 0.0% (0/1)	moderate: 0.0% (0/4)	moderate: 100.0% (2/2)
		mild: 0.0% (0/11)	mild: 0.0% (0/1)	mild: 25.0% (1/4)	mld: 0.0% (0/2)
	epilepsy	72.7% (8/11)	100.0% (1/1)	75.0% (3/4)	50.0% (1/2)
	estimated level of ID	severe: 90.9% (10/11)	severe: 100.0% (1/1)	severe: 100.0% (4/4)	severe: 0.0% (0/2)
		moderate: 9.1% (1/11)	moderate: 0.0% (0/1)	moderate: 0.0% (0/4)	moderate: 100.0% (2/2)
	ataxia	66.7% (6/9)	100.0% (1/1)	0.0% (0/2)	100.0% (1/1)
	hypotonia	100.0% (11/11)	100.0% (1/1)	75.0% (3/4)	100.0% (2/2)
	language ability	non-verbal: 63.6% (7/11)	non-verbal: 100.0% (1/1)	non-verbal: 100.0% (4/4)	non-verbal: 0.0% (0/2)
		few words: 27.3% (3/11)	few words: 0.0% (0/1)	few words: 0.0% (0/4)	few words: 0.0% (0/2)
		simple sentences: 9.1% (1/11)	simple sentences: 0.0% (0/1)	simple sentences: 0.0% (0/4)	simple sentences: 100.0% (2/2)
	sleep disturbances	40.0% (4/10)	0.0% (0/1)	0.0% (0/4)	N/A
Vision	ophthalmologic/vision abnormalities	72.7% (8/11)	100.0% (1/1)	0.0% (0/2)	100.0% (1/1)
Gastrointestinal issues	constipation	70.0% (7/10)	100.0% (1/1)	50.0% (2/4)	N/A
	feeding difficulties	63.6% (7/11)	100.0% (1/1)	25.0% (1/4)	0.0% (0/2)
	gastroesophageal reflux	20.0% (2/10)	0.0% (0/1)	0.0% (0/4)	N/A
	growth problems	81.8% (9/11)	0.0% (0/1)	25.0% (1/4)	100.0% (2/2)
	low weight	81.8% (9/11)	0.0% (0/1)	25.0% (1/4)	100.0% (2/2)
Bone/skeletal anomalies	bone/skeletal anomalies	27.3% (3/11)	0.0% (0/1)	0.0% (0/4)	50.0% (1/2)
	hip dysplasia	30.0% (3/10)	0.0% (0/1)	0.0% (0/4)	0.0% (0/2)
	scoliosis	9.1% (1/11)	100.0% (1/1)	0.0% (0/4)	0.0% (0/2)
Other systemic issues	acrocyanosis	18.2% (2/11)	0.0% (0/1)	0.0% (0/4)	0.0% (0/2)
	blood count abnormalities	0.0% (0/9)	0.0% (0/1)	0.0% (0/4)	0.0% (0/2)
	cardiac abnormalities	9.1% (1/11)	0.0% (0/1)	0.0% (0/4)	0.0% (0/2)
	cutaneous abnormalities	27.3% (3/11)	100.0% (1/1)	0.0% (0/4)	100.0% (2/2)
	digital anomaly	30.0% (3/10)	0.0% (0/1)	0.0% (0/4)	100.0% (2/2)
	hearing loss	0.0% (0/11)	0.0% (0/1)	0.0% (0/4)	0.0% (0/2)
	join hyperlaxity	36.4% (4/11)	0.0% (0/1)	0.0% (0/4)	N/A
	renal/genitourinary abnormalities	9.1% (1/11)	0.0% (0/1)	0.0% (0/4)	100.0% (2/2)
	teeth/dental anomalies	20.0% (2/10)	100.0% (1/1)	0.0% (0/4)	100.0% (1/1)
Other core features	dysmorphic facial features	90.9% (10/11)	100.0% (1/1)	50.0% (2/4)	100.0% (2/2)
	sialorrhea	80.0% (8/10)	100.0% (1/1)	0.0% (0/2)	N/A
	macrocephaly (*RNU5B-1*)	–	100.0% (1/1)	–	–
	marfanoid habitus (*RNU5B-1*)	–	0.0% (0/1)	–	–
	pectus excavatum (*RNU5B-1*)	–	100.0% (1/1)	–	–
	hyperventilation (*RNU2-2*)	–	–	0.0% (0/4)	–
	brachydactyly (*RNU4ATAC*)	–	–	–	50.0% (1/2)
	clinodactyly (*RNU4ATAC*)	–	–	–	50.0% (1/2)
	endocrine abnormalities (*RNU4ATAC*)	–	–	–	100.0% (2/2)
	frequent infections (*RNU4ATAC*)	–	–	–	100.0% (2/2)
	genital abnormalities (*RNU4ATAC*)	–	–	–	100.0% (2/2)
	joint contractures (*RNU4ATAC*)	–	–	–	0.0% (0/2)
	micrognathia (*RNU4ATAC*)	–	–	–	50.0% (1/2)
	transverse palmar creases (*RNU4ATAC*)	–	–	–	100.0% (2/2)

a Patient 12 (*RNU4-2*; n.18_19insA) presented no representative symptoms of ReNU syndrome other than retinitis pigmentosa and was thus excluded.

In one patient, a previously reported recurrent pathogenic *de novo RNU5B-1* variant, n.42_43insA (NR_002757.3), located in the U5 5′ loop I, was identified (Figure 1C; Tables 1 and 2). Although this patient shared multiple phenotypic features with individuals harboring *RNU4-2* and *RNU2-2* variants, relative macrocephaly and pectus excavatum were distinctive characteristics. Marfanoid habitus was not observed. Similar clinical features, including macrocephaly, pectus excavatum, and marfanoid habitus, have been reported previously in individuals with *RNU5B-1* variants.^8^^,^^9^

Among patients with *RNU2-2* variants, one harbored the highly recurrent *de novo* n.4G>A (NR_199791.1) variant. Another patient carried compound heterozygous variants (n.58T>G and n.66A>C), each inherited from a different parent, consistent with recent reports describing an autosomal recessive inheritance pattern for *RNU2-2*.^12^^,^^13^ In addition, two affected siblings were identified who carried compound heterozygous *RNU2-2* variants, n.100T>G and n.155C>G. Thus, a total of four patients with *RNU2-2*-associated disease were included in this study (Figure 1D; Tables 1 and 2). Consistent with previous studies, patients with *RNU2-2* variants exhibited phenotypic overlap with ReNU syndrome and uniformly developed intractable epilepsy or were suspected of it. The age at epilepsy onset ranged from 21 months to 3 years. One patient (patient 14) was subsequently diagnosed with Lennox-Gastaut syndrome at 14 years of age. Ventilatory abnormalities, such as hyperventilation, were not consistently observed (Document S1).

Both patients with *RNU4ATAC* variants harbored biallelic variants. One patient carried compound heterozygous variants, n.13C>T and n.13C>G (NR_023343.3), whereas the other carried n.13C>T and n.119T>C (Figure 1E; Tables 1 and 2). Both combinations are rare. Both n.13 variants have been reported previously, with n.13C>T described frequently^20^ and n.13C>G reported in three prior cases,^23^^,^^24^^,^^25^ whereas the n.119T>C variant has not been described previously and is reported here for the first time in a symptomatic individual. This novel variant is located within the Sm protein-binding site of *RNU4ATAC,* a region frequently implicated in Roifman Syndrome-associated pathogenic variants,^26^^,^^27^^,^^28^^,^^29^ often in combination with n.13C>T. Whereas *RNU4-2*, *RNU2-2*, and *RNU5B-1* are components of the major (U2-type) spliceosome, *RNU4ATAC* is a constituent of the minor (U12-type) spliceosome. Disorders associated with U2 spliceosomal components predominantly manifest as NDDs, whereas biallelic *RNU4ATAC* variants cause a spectrum of autosomal recessive multisystem developmental disorders, including microcephalic osteodysplastic primordial dwarfism type 1 (MOPD I), Lowry-Wood syndrome (LWS), Roifman syndrome, and Joubert syndrome, which are characterized by variable combinations of growth retardation, microcephaly, skeletal abnormalities, neurodevelopmental impairment, and immune dysfunction. The two probands identified in this study exhibited clinical features most consistent with Roifman Syndrome, including moderate intellectual disability, growth failure, and recurrent respiratory infections. Notably, both patients developed Guillain-Barré Syndrome at 4 years of age. In addition, patient 18 presented with poorly controlled fulminant type 1 diabetes mellitus, a feature recently associated with autoimmune dysfunction in individuals harboring pathogenic *RNU4ATAC* variants^5^ (Document S1).

Previous studies have reported that, with few exceptions, *de novo* autosomal dominant pathogenic variants in snRNA genes preferentially arise on the maternal allele.^3^^,^^7^^,^^9^ Although haplotype phasing using heterozygous variants can, in principle, determine parental origin, this approach is rarely feasible with short-read sequencing because pathogenic variants and informative parental heterozygous variants are seldom captured within the same read. To overcome this limitation, we amplified PCR products varying from several hundred to several thousand base pairs encompassing heterozygous variants and performed long-read nanopore sequencing to determine the parental origin of *de novo* variants. As shown in Figure 1F, haplotype phasing demonstrated that all *de novo* variants analyzed in this study originated from the maternal allele (Table 1).

### Construction and assessment of snRNA-extended WES

Target snRNA genes were first identified through HUGO Gene Nomenclature Committee (HGNC) by filtering for approved snRNA gene symbols that were not annotated as pseudogenes, yielding 51 genes. Genomic positional information was then obtained from the Ensembl annotation file. Because positional information was unavailable only for RNVU1-20, this was excluded, and a final set of 50 snRNAs was selected as targets. Custom capture probes targeting these genes were designed (see STAR Methods). Capture probes were successfully generated for all 50 snRNA genes (Table S3) and mixed at equimolar concentrations with standard exome probes for subsequent evaluation. Hereafter, this combined probe set is referred to as the snRNA-extended WES probes.

**Figure 2:**
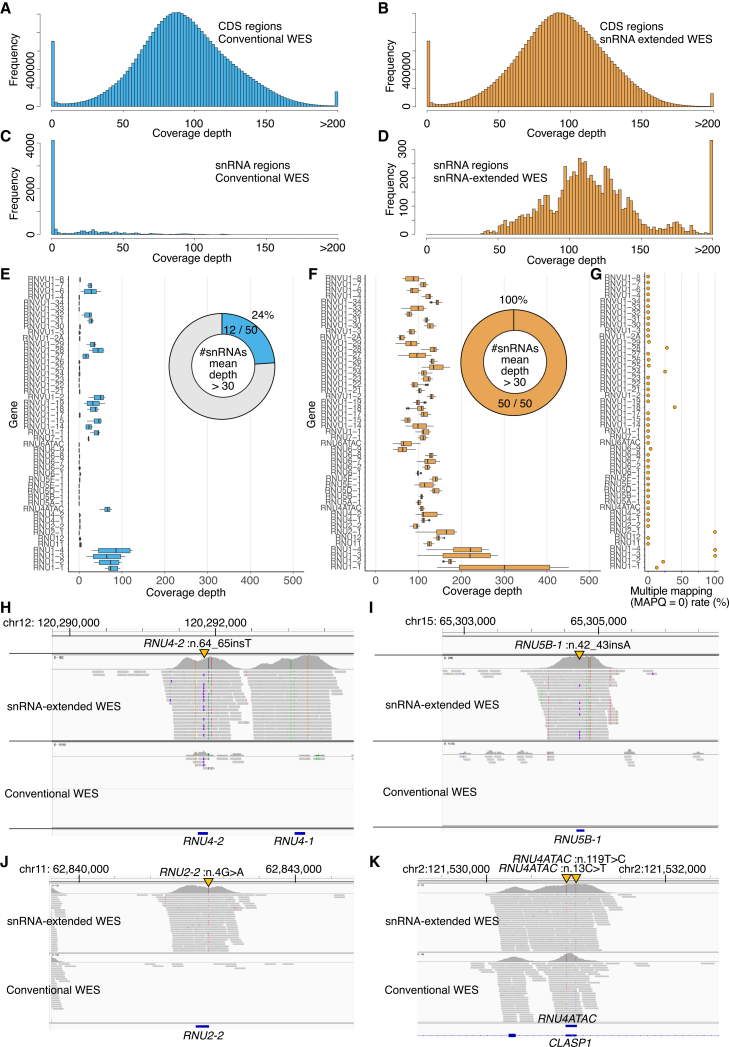
Coverage comparison between conventional WES and snRNA-extended WES (A and B) Histograms showing coverage depth across all CDS regions obtained by conventional WES (A) and snRNA-extended WES (B). Data shown in (A) through (F) were generated from the HG002 sample using either conventional WES or snRNA-extended WES, with sequencing output normalized to 9 Gb. All samples were run on the same platform. (C and D) Histograms showing coverage depth across all 50 targeted snRNA regions obtained by conventional WES (C) and snRNA-extended WES (D). (E and F) Boxplots showing coverage depth for each of the 50 snRNA genes obtained by conventional WES (E) and snRNA-extended WES (F). Center line, median; box limits, upper and lower quartiles; whiskers, 1.5× interquartile range (IQR); dots, values beyond 1.5× IQR. Pie charts in the upper right indicate the proportion of snRNA genes with a mean coverage depth greater than 30. (G) IGV images comparing conventional WES and snRNA-extended WES performed on genomic DNA from patient 2 harboring an *RNU4-2* variant. (H) IGV images comparing conventional WES and snRNA-extended WES performed on genomic DNA from patient 13 harboring an *RNU5B-1* variant. (I) IGV images comparing conventional WES and snRNA-extended WES performed on genomic DNA from patient 14 harboring an *RNU2-2* variant. (J) IGV images comparing conventional WES and snRNA-extended WES performed on genomic DNA from patient 19 harboring an *RNU4ATAC* variant.

To assess the performance of snRNA-extended WES probes, we first applied them to the benchmark reference samples HG001 and HG002. snRNA-extended WES and conventional WES without snRNA-targeted probes were compared using equal amounts of sequencing data (9 Gb per sample). Relative to conventional WES, snRNA-extended WES yielded a substantially higher number of sequencing reads across snRNA gene regions (Figures 2A–2F and S2; Table S4). Across all 50 snRNA genes, including those previously reported to harbor disease-causing variants, snRNA-extended WES achieved a mean coverage depth of 106.00 and a median coverage depth of 106.

To evaluate the specificity of read mapping to snRNA genes, we quantified the proportion of multi-mapped reads at each locus by calculating the fraction of reads with a mapping quality score (MAPQ) of 0. Among the 50 targeted RNU genes, three loci—*RNU1-2*, *RNU1-3*, and *RNU2-1*—showed an extremely high proportion of multi-mapped reads (∼100%), indicating that reliable variant calling is not feasible at these loci (Figures 2G and S2G). In contrast, many RNU genes exhibited predominantly unique read mapping despite their high sequence similarity. For example, *RNU4-1* and *RNU4-2* differ by only 4 nucleotides out of 141 bases, and members of the RNU6 gene family (*RNU6-1*, *RNU6-2*, *RNU6-7*, *RNU6-8*, and *RNU6-9*) have completely identical sequences across all 107 bases. Nevertheless, reads mapped to these loci were largely uniquely aligned, as demonstrated by low multiple mapping rates (Figures 2H and S3).

We next evaluated whether the addition of custom probes targeting snRNA genes adversely affected capture efficiency or variant calling performance in standard exonic regions targeted by WES. Using genomic DNA from the HG001 and HG002 samples, we compared three datasets: conventional WES, replicate conventional WES, and snRNA-extended WES. To ensure a fair comparison, sequencing output (fastq file) was normalized to 9 Gb for all datasets prior to analysis. First, we confirmed that there were no appreciable differences in mean coverage across autosomal CDS regions included in the target, nor in the proportions of target bases covered at ≥10×, ≥20×, and ≥30× (Table S5). Furthermore, metrics reflecting coverage uniformity and capture performance—including fold-80 base penalty, on-target rate, duplicate rate, and exon-level correlation coefficients—were comparable between snRNA-extended WES and conventional WES, and were within the range observed between conventional WES replicates, indicating no detectable impact of adding snRNA probes (Table S5). Next, for both HG001 and HG002, we assessed variant calling performance using the genome in a bottle (GIAB) high-confidence variant sets as a benchmark.^30^ Evaluation based on precision, recall, and F1 score demonstrated that the addition of snRNA probes did not adversely affect variant detection performance (Table S5).

Finally, we applied snRNA-extended WES to patient samples in which pathogenic snRNA variants had been identified by WGS. This approach robustly captured the *RNU4-2 RNU5B-1*, *RNU2-2*, and *RNU4ATAC* variants, demonstrating its effectiveness for detecting disease-associated snRNA variants (Figures 2H–2K). *RNU4ATAC* was also captured and sequenced by conventional WES because it overlaps with the *CLASP1* gene (Figure 2K).

## Discussion

This study demonstrates the practical utility of extending conventional WES to include disease-relevant snRNA loci. By incorporating targeted capture probes for known pathogenic snRNA genes, we show that this approach enables detection of disease-causing variants that are otherwise missed by standard WES, while preserving the advantages of exome-based testing. In the present study, the snRNA targets comprised only 50 genes (7,399 bases), a total target size smaller than that of mitochondrial DNA (mtDNA) and which accounts for less than 0.03% of the total CDS regions captured by conventional WES. Accordingly, the addition of snRNA probes does not require an increase in total sequencing output, circumventing the substantial computing and storage costs associated with WGS. From a cost-efficiency perspective, snRNA-extended WES provides a substantial advantage over WGS. Although sequencing costs vary depending on the platform, reagent selection, analytical workflow, and geographic setting, representative estimates from Japan indicate that standard WES costs approximately $200 per sample, whereas WGS costs approximately $400 per sample. In this context, the additional probe set designed to capture snRNA genes in our study resulted in only a modest increase of approximately $7 per sample (+3.5%) due to its small size (7,399 bp). Also importantly, the experimental workflow remains essentially unchanged, requiring only the addition of the snRNA-specific probe mixture to the conventional exome probe solution, with no further modifications to the protocol. As the total sequencing output is not increased and no additional sequencing reagents or throughput are required, the overall cost structure of conventional WES is preserved. Thus, compared with performing WGS upfront, snRNA-extended WES yields an approximate cost saving of $193 per sample. Under a fixed budget, this would enable the analysis of approximately 1.9-fold more samples than WGS. These findings suggest that snRNA-extended WES represents a cost-effective intermediate strategy, particularly in resource-constrained settings, enabling an approximately 1% increase in diagnostic yield while maintaining scalability in clinical practice. By improving both accessibility and affordability, snRNA-extended WES has the potential to expand the scope of molecular diagnostic testing and facilitate genetic diagnosis for patients who remain undiagnosed worldwide.

Recent discoveries identifying *RNU4-2* and other snRNA genes as causes of NDDs exemplify how advances in large-scale sequencing and improved analytical approaches have accelerated the identification of disease-causing genes. Early reports suggested that the identification of pathogenic variants in *RNU4-2* and *RNU2-2* could explain approximately 0.5% of previously unsolved NDD cases.^3^^,^^7^ In the present study, by expanding both the range of target disorders and the spectrum of snRNA genes analyzed, we identified pathogenic variants in 1.2% of previously genetically unsolved cases. These findings suggest that the clinical relevance and diagnostic contribution of snRNA analysis are likely to increase further as additional disease-associated snRNA genes are recognized. They also highlight the value of expanding access to genomic diagnostics though efficient, affordable tools. Cost-effective adaptations, such as our extended-exome approach, can help bridge the gap between research discoveries and clinical practice by enabling more rapid identification of pathogenic variants in patients who remain undiagnosed. Ultimately, such advances may shorten the diagnostic odyssey experienced by many patients.

Among snRNAs, *RNU4ATAC* was recognized relatively early as a cause of autosomal recessive disease.^18^^,^^19^ Although understanding of *RNU4ATAC*-associated disorders has advanced substantially in recent years, progress toward disease-modifying or curative therapies has remained limited. Recent studies have demonstrated that immunoglobulin replacement therapy can reduce infectious burden in patients with Roifman syndrome.^24^^,^^31^ Although such interventions do not address the underlying molecular defect, they may represent a pragmatic approach to disease management by preventing severe and potentially life-altering infection-related complications, such as Guillain-Barré syndrome, as observed in the patients described in this study.

Consistent with previous reports, all *de novo* autosomal dominant variants identified in our cohort arose on the maternal allele. Although this parent-of-origin bias has been discussed previously,^3^^,^^7^^,^^9^ no definitive mechanistic explanation has yet been established. One possibility is negative selection against pathogenic variants during spermatogenesis. Alternatively, paternal splicing dysfunction during early embryogenesis may result in embryonic lethality, thereby preventing the observation of paternally derived variants. If the latter hypothesis is correct, large-scale WGS studies focused on embryonic lethality^32^ may uncover pathogenic *RNU4-2* variants arising on the paternal allele, despite substantial technical and ethical challenges involved.

snRNAs constitute a class of non-coding RNA genes that play essential roles in pre-mRNA splicing; however, the clinical phenotypes associated with pathogenic variants vary substantially depending on the specific snRNA gene involved.^33^ Moreover, accumulating evidence indicates that clear genotype-phenotype correlations exist not only between different snRNA genes but also within individual snRNA loci. For example, in *RNU4-2*, pathogenic variants located within the T-loop region (n.63–67) are consistently associated with more severe neurodevelopmental phenotypes, whereas variants affecting the Stem III region (n.72–79) tend to result in comparatively milder clinical presentations, as reported in multiple independent studies.^9^^,^^34^ Importantly, even among variants with the same mode of inheritance, phenotypic heterogeneity can be pronounced. In our cohort, autosomal dominant variants affecting the T-loop region of *RNU4-2* (patients 1–11) were associated with typical NDD phenotypes, whereas a distinct autosomal dominant variant, n.18_19insA (patient 12), was primarily associated with retinitis pigmentosa, highlighting a marked divergence in clinical manifestations depending on the underlying genotype. These observations strongly suggest that RNA secondary structure and the functional roles of specific structural elements within the snRNA molecule critically influence disease phenotype and may be implicated in tissue-specific pathogenicity. Furthermore, different inheritance patterns within the same snRNA gene can also give rise to distinct phenotypic spectra. For instance, autosomal recessive *RNU4-2*-associated disorders have been reported to exhibit clinical features partially distinct from the dominant form, including perivascular space enlargement and cerebellar atrophy.^10^^,^^11^ Similar reports of clinically distinct phenotypes have been made for autosomal recessive *RNU2-2*-associated disorders.^14^ Although recent studies have demonstrated that saturation genome editing is a powerful approach for assessing the pathogenicity of novel snRNA variants,^10^ continued accumulation of well-characterized clinical cases, including those reported in the present study, will be essential for further expanding the phenotypic spectrum and refining our understanding of RNA structure-based genotype-phenotype correlations in snRNA-associated disorders.

In conclusion, our study demonstrates that extending exome capture designs to include disease-associated snRNA genes provides a practical and economical means of improving molecular diagnostic yield. As the catalog of pathogenic non-coding variants continues to expand, such flexible sequencing strategies may bridge the gap between research discoveries and routine clinical diagnostics, ultimately facilitating earlier and more precise genetic diagnoses.

### Limitations of the study

Nevertheless, this study has several limitations. First, the current capture design targeted only 50 snRNA genes approved by the HGNC. As illustrated by the case of *RNU2-2*, which was annotated as a pseudogene (*RNU2-2P*) until human genome build 37 and subsequently reannotated as *RNU2-2*, additional disease-associated snRNA genes are likely to be identified in the future. Accordingly, periodic updates of capture probe designs will be required to maintain comprehensive diagnostic coverage.

In addition, several snRNA genes share highly similar genomic sequences, which could potentially reduce probe specificity. Nevertheless, our results indicate that many loci can be reliably interrogated using snRNA-extended WES. One plausible explanation is that, although the snRNA genes themselves are short and highly similar in sequence, their flanking genomic regions are unique within the genome. Sequencing reads that span these unique regions may enable unambiguous mapping, thereby potentially mitigating the impact of sequence similarity on probe specificity and variant calling accuracy. However, our analysis also highlights that a subset of loci, such as *RNU1-2*, *RNU1-3*, and *RNU2-1*, remain problematic due to pervasive multi-mapping, indicating that caution is required when interpreting variants in these regions. Notably, all snRNA genes previously reported to harbor pathogenic or likely pathogenic variants were sufficiently covered and did not exhibit issues related to sequence redundancy during probe design or read mapping. Collectively, these findings support the overall reliability of our approach while delineating its limitations in highly repetitive or identical loci. While this proof-of-concept study demonstrates technical feasibility, validation in larger cohorts will be necessary to rigorously assess sensitivity and specificity in real-world diagnostic settings.

## Resource availability

### Lead contact

Further information and requests should be directed to the lead contact, Fuyuki Miya (fmiya@keio.jp).

### Materials availability

All materials relevant to this manuscript are available from the lead contact with a completed materials transfer agreement.

### Data and code availability

The HG001 and HG002 sequencing data in this study are freely available in the DDBJ Sequencing Read Archive (SRA) under the accession number DRR792288, DRR792289, DRR1045491, DRR1045492, and DRR1045493 (project no. PRJDB37987). No unique code was generated for this manuscript. Other data reported in this paper will be shared by the lead contact upon request.

## Acknowledgments

This work was supported in part by the 10.13039/100009619Japan Agency for Medical Research and Development (10.13039/100009619AMED) under grant numbers grant number JP25ek0109760 (to Kenjiro Kosaki), JP25ek0109672 (to F.M. and Kenjiro Kosaki), and JP25gn0110083 (to T.T. and Kenjiro Kosaki), and JP25ek0109617 (to Kenjiro Kosaki). This work was also supported by 10.13039/501100001691JSPS KAKENHI grant number 24K14705 (to F.M.), and JST BOOST Japan grant number JPMJBY24H3 (to F.M.). We thank the patients and their families for their consent to the publication of this report. We also thank S. Kumagai, K. Ohta, Y. Ohbayasi, K. Tsukue, Y. Yamamoto, H. Nakamura, T. Hayashida, K. Misu, I. Ono, Y. Wada, M. Yoshimura, K. Sada, M. Noguchi, C. Yoshida, T. Taya, and Y. Uemura for technical support in this study.

## Author contributions

Y.N., data curation, formal analysis, investigation, visualization, writing − original draft and editing; H. Suzuki, data curation, formal analysis, investigation, writing − original draft; Y.K., investigation, writing − review and editing; H. Yoshihashi, investigation, writing − review and editing; N.O., investigation, writing − review and editing; A.K., investigation, writing − review and editing; R.K., investigation, writing − review and editing; K. Kashimada, investigation, writing − review and editing; T.K., investigation, writing − review and editing; M.-K.T., investigation, writing − review and editing; S.-K.T., investigation, writing − review and editing; M.R., investigation, writing − review and editing; T.E., investigation, writing − review and editing; H. Suzumura, investigation, writing − review and editing; T.Y., investigation, writing − review and editing; S.K., investigation, writing − review and editing; S.M., investigation, writing − review and editing; M.I., investigation, writing − review and editing; N.N., investigation, writing − review and editing; M.M., investigation, writing − review and editing; E.O., investigation, writing − review and editing; HYagi, investigation, writing − review and editing; M.Y., investigation, writing − review and editing; E.Q., data curation, writing − review and editing; D.N., investigation, writing − review and editing; T.T., conceptualization, investigation, funding acquisition, writing − review and editing; K. Kosaki, conceptualization, funding acquisition, supervision, writing − review and editing. F.M., conceptualization, funding acquisition, supervision, data curation, visualization, formal analysis, investigation, writing − original draft, review, and editing.

## Declaration of interests

The authors declare that the research was conducted in the absence of any commercial or financial relationships that could be construed as a potential conflict of interest.

## Declaration of generative AI and AI-assisted technologies in the writing process

No generative AI or AI-assisted technologies were used in the preparation of this manuscript.

## STAR★Methods

### Key resources table

**Table:** 

REAGENT or RESOURCE	SOURCE	IDENTIFIER
**Biological samples**		

HG001 (NA12878) DNA	Coriell Institute	NA12878
HG002 (NA24385) DNA	Coriell Institute	NA24385

**Critical commercial assays**		

Twist KCMG Exome Nanbyome 2024, 12 Reaction capture probes	Twist Bioscience	Cat# 110751
snRNA Custom Probes (Twist Bioscience)	This study	N/A
PromethION Flow Cell	Oxford Nanopore Technologies (ONT)	Cat# FLO-PRO114M
Ligation Sequencing Kit V14	ONT	Cat #SQK-LSK114
Flow Cell Wash Kit	ONT	Cat# EXP-WSH004

**Deposited data**		

WES data (FASTQ files) of the HG001 sample generated with and without the inclusion of capture probes for snRNA	This study	DDBJ SRA: DRR1045491; DRR1045492; DRR1045493 (Project No. PRJDB37987)
WES data (FASTQ files) of the HG002 sample generated with and without the inclusion of capture probes for snRNA	This study	DDBJ SRA: DRR792288; DRR792289 (Project No. PRJDB37987)
Benchmark High-confidence variant calls for HG001	Genome in a Bottle (GIAB)	https://ftp-trace.ncbi.nlm.nih.gov/ReferenceSamples/giab/release/NA12878_HG001/NISTv4.2.1/GRCh38/
Benchmark High-confidence variant calls for HG002	Genome in a Bottle (GIAB)	https://ftp-trace.ncbi.nlm.nih.gov/ReferenceSamples/giab/release/AshkenazimTrio/HG002_NA24385_son/NISTv4.2.1/GRCh38/

**Oligonucleotides**		

The PCR primer sequences used for Sanger sequencing validation and haplotype determination are provided in Table S2.	This study	N/A

**Software and algorithms**		

DRAGEN v4.3	Illumina	https://help.dragen.illumina.com/dragen-v4.3
ANNOVAR v2025Jul29	Wang et al.^37^	RRID: SCR_012821https://annovar.openbioinformatics.org/
dbNSFP v5.3.1a	Liu et al.^38^	RRID: SCR_005178 https://www.dbnsfp.org
HGMD Professional v2025.4	Qiagen	https://digitalinsights.qiagen.com/products-overview/clinical-insights-portfolio/human-gene-mutation-database/
MinKNOW v25.09.16	ONT	https://nanoporetech.com/software/devices/p2-solo/software
Dorado v0.9.1	ONT	RRID: SCR_025883https://nanoporetech.com/ja/software/other/dorado
Dorado model (dna_r10.4.1_e8.2_400bps_sup@v5.0.0)	ONT	https://software-docs.nanoporetech.com/dorado/latest/models/downloader/
minimap2 v2.28	Li et al.^39^	RRID: SCR_018550 https://github.com/lh3/minimap2
PEPPER–Margin–DeepVariant r0.8	Shafin et al.^40^	https://github.com/kishwarshafin/pepper
Genome Analysis Toolkit (GATK) v4.6.2.0	Broad Institute	RRID: SCR_001876 https://gatk.broadinstitute.org
SeqKit2 v2.9.0	Shen et al.^41^	RRID: SCR_018926https://github.com/shenwei356/seqkit
R v4.5.1	R Foundation for Statistical Computing	RRID: SCR_001905 https://www.r-project.org
RStudio v2024.12.0+467	Posit Software, PBC	RRID: SCR_000432https://posit.co/products/open-source/rstudio/
Bioconductor v3.2.2	Bioconductor Project	RRID: SCR_006442https://bioconductor.org
trackViewer v1.41.6	Bioconductor	RRID: SCR_027743https://bioconductor.org/packages/trackViewer
Integrated Genomics Viewer (IGV) v2.19.1	Broad Institute	RRID: SCR_011793https://igv.org
bedtools v2.31.1	Quinlan and Hall^42^	RRID: SCR_006646https://bedtools.readthedocs.io
ggplot2 v4.0.1	Wickham^43^	RRID:SCR_014601https://ggplot2.tidyverse.org
hap.py v0.3.15	Illumina	https://github.com/Illumina/hap.py

### Experimental model and study participant details

#### Patients and participants

Patients were recruited through the Initiative on Rare and Undiagnosed Diseases (IRUD)^44^ and the Precise and Rapid Genetic Diagnosis and Treatability for Infants (Priority-i) project.^45^ Peripheral blood samples were obtained from patients and their parents, and genomic DNA was extracted using standard protocols. There were no restrictions on participants’ age, sex, or ancestry; however, most participants were pediatric patients with congenital disorders suspected to have a genetic etiology, and DNA sequencing of all participants was performed in Japan.

The study protocol was approved by the Ethics Committees of Keio University School of Medicine (approval No. 20110262 and 20211032). All procedures were conducted in accordance with the approved guidelines. Written informed consent was obtained from all participants or their legal guardians prior to enrollment.

#### Cell DNAs

Genomic DNA from HG001 (NA12878) and HG002 (NA24385) DNAs were purchased from the Coriell Institute and used as a benchmark reference sample^46^ for sequencing and data generation.

### Method details

#### Whole-genome sequencing and the analysis

Sequencing libraries for short-read whole-genome sequencing (WGS) were prepared from genomic DNA using the TruSeq DNA PCR-Free Kit or the Illumina DNA PCR-Free Prep Kit (Illumina) according to the manufacturers’ instructions. WGS was performed on the Illumina NovaSeq 6000 or NovaSeq X/X Plus platforms.

WGS data were analyzed using Illumina DRAGEN (v4.3) following the manufacturer’s recommended pipelines. Single-nucleotide variants (SNVs), short insertions and deletions (indels), structural variants (SVs), and repeat expansions detected by DRAGEN were used for downstream analyses. Variants were filtered to those with an allele frequency <0.5% in gnomAD^35^ and jMorp^36^ (a Japanese population allele frequency database) and with a homozygous allele count <2. Candidate disease-associated variants were subsequently evaluated. Gene-based annotation was performed using ANNOVAR (https://annovar.openbioinformatics.org/), and in silico pathogenicity prediction scores were annotated using dbNSFP (v5.3.1; https://www.dbnsfp.org). Known disease-associated variants databases, including ClinVar (https://www.ncbi.nlm.nih.gov/clinvar/) and HGMD Professional (Qiagen) were consulted. When parental DNA was available, trio-based analyses were performed, and candidate pathogenic variants were filtered according to the expected mode of inheritance.

#### Long-read sequencing analysis

Long-read sequencing (LRS) was performed using PromethION flow cells (R10.4.1; Oxford Nanopore Technologies (ONT)) as previously described.^47^ Sequencing libraries were prepared from PCR-amplified products using the Ligation Sequencing Kit V14 (ONT) and sequenced on the PromethION 2 (P2) Solo platform (ONT). Sequencing runs were controlled using MinKNOW software (v25.09.16; ONT) with a sampling frequency of 5 kHz. After each sequencing run, flow cells were washed using the Flow Cell Wash Kit (ONT) according to the manufacturers’ protocol and reused for subsequent runs. Basecalling was performed using Dorado (v0.9.1; ONT) with a super-accuracy model (dna_r10.4.1_e8.2_400bps_sup@v5.0.0). Basecalled reads were aligned to the human reference genome (GRCh38/hg38) using minimap2^39^ (v2.28). Haplotype-aware variant calling was performed using PEPPER–Margin–DeepVariant^40^ (v0.8.5).

#### Custom capture probes for snRNA genes

Official gene symbols encoding functional snRNAs were obtained from the HUGO Gene Nomenclature Committee (HGNC; https://www.genenames.org/). Gene information was retrieved from HGNC in September 2025 by applying the advanced filtering gd_locus_type = “RNA, small nuclear” and restricting results to approved gene symbols, and selecting only gene symbols without pseudogene annotation. Genomic coordinates for snRNA genes were obtained from Ensembl (release 116; ftp://ftp.ensembl.org/pub/release-116/vertebrates/gtf/homo_sapiens/Homo_sapiens.GRCh38.116.chr.gtf.gz). Target snRNA genomic coordinates were defined in BED format and provided in Table S3. To ensure robust capture, genomic coordinates were extended by 250 base pairs upstream and downstream prior to probe design. Custom capture probes targeting snRNA genes were designed and synthesized by Twist Bioscience. In the probe design, 120-mer probes were designed to avoid repetitive elements such as SINEs and LINEs, to preferentially select sequences with maximal uniqueness, and above all, to ensure overlapping probe coverage across the target regions.

#### Extended whole-exome sequencing

Extended whole-exome sequencing (WES) was conducted as described previously.^22^ Conventional WES libraries in this study were generated using a custom-designed panel termed “Nanbyome,” based on the Twist Exome 2.0 plus Comprehensive Exome spike-in probe set (Twist Bioscience) supplemented with custom probes targeting intronic and untranslated regions (UTRs) of selected hereditary disease–associated genes, disease-related repeat regions, and the mitochondrial genome. In the present study, additional custom probes specific for snRNA genes were included at equimolar concentrations with probes targeting protein-coding exonic regions, generating the snRNA-extended WES design. Library preparation was preformed using the Twist Library Preparation EF Kit 2.0 according to the manufacturers’ instructions, and sequencing was conducted on the DNBSEQ-G50 platform (MGI).

WES data were analyzed as described previously.^22^^,^^48^ The variant calling was performed using GATK (v4.6.2.0) following the GATK Best Practices workflow.^49^ When normalization of sequencing depth across samples was required for validation, SeqKit2^41^ was used. Filtering criteria for pathogenic variants were identical to those applied in the WGS analysis described above.

### Quantification and statistical analysis

All data preprocessing, statistical analyses, and plot generation were performed using R (v4.5.1) within the RStudio environment. In box-and-whisker plots, the center line represents the median; the box limits indicate the upper and lower quartiles; whiskers extend to 1.5× the interquartile range (IQR); and dots denote values beyond 1.5× the IQR.

For data visualization, lollipop plots were generated using the lolliplot function in the trackViewer package (v1.41.6) within Bioconductor (v3.2.2). Sequencing reads and overlaid variants were visualized using the Integrated Genomics Viewer (IGV; v2.19.1). Coverage depth of the target regions was assessed using bedtools^42^ (v2.31.1). Histograms were generated using the hist function in R, and box plots were created using ggplot2^43^ (v4.0.1). For the HG001 and HG002 samples, comparisons with the GIAB high-confidence variant calls (v4.2.1) were performed using hap.py (v0.3.15).^30^ All figures were assembled using Adobe Illustrator (v30.1, 2026).

## Supplemental information

Document S1. Figures S1–S3

Table S1. The variants used to generate the lollipop plot in Figure 1 and the publications reporting these variants

Table S2. PCR primer sets for Sanger sequencing validation and determines haplotypes

Table S3. Targeted snRNA genes in BED file (0-based) format

Table S4. Sequencing depth of snRNA genes in six sample for conventional WES and snRNA-extended WES after normalizing the sequencing yield to 9 Gb

Table S5. Coverage metrics and benchmarking of variant calls against GIAB high-confidence variants for conventional and snRNA-extended WES in HG001 and HG002
