# Supplementary material for: Whole-genome discovery of pathogenic snRNA variants and efficient extended-exome screening
Source: iScience. 2026 Jul 16;29(8):116814. doi: 10.1016/j.isci.2026.116814 (PMC13400782; doi:10.1016/j.isci.2026.116814)
Supplement: Document S1. Figures S1–S3 [file mmc1.pdf]

iScience, Volume *29*

Supplemental information

Whole-genome discovery of pathogenic snRNA variants and efficient extended-exome screening

Yuka Nakano, Hisato Suzuki, Yukiko Kuroda, Hiroshi Yoshihashi, Nobuhiko Okamoto, Akane Kondo, Rika Kosaki, Kenichi Kashimada, Toshihide Kurihara, Meow-Keong Thong, Sok-Kun Tae, Mazlan Rifhan, Takashi Enokizono, Hiroshi Suzumura, Takeshi Yoshida, Shinji Kosugi, Seiji Mizuno, Mie Inaba, Natsuki Nakamura, Mayumi Matsufuji, Eri Ogawa, Hitomi Yagi, Mamiko Yamada, Emi Qian, Daisuke Nakato, Toshiki Takenouchi, Kenjiro Kosaki, and Fuyuki Miya

Document S1. Clinical phenotypes of patients in whom pathogenic variants in snRNA were identified

Patient 1

11-year-old female (*RNU4-2*, n.64_65insT de novo)

The patient was born at 38+5 weeks of gestation with a birth weight of 2,500 g, without perinatal asphyxia. Physical examination revealed microcephaly, a triangular-shaped mouth, and a prominent nasal bridge.

Developmental milestones included head control at 6 months and independent sitting at 10 months. Thereafter, motor development was markedly delayed; at 3 years of age, rolling over remained incomplete and she was unable to stand with support. At 11 years 8 months of age (at the time of diagnosis), she had not yet achieved rolling over and had no meaningful words, consistent with global developmental delay. She has epilepsy, and seizures were currently controlled with perampanel, levetiracetam, rufinamide. Brain MRI demonstrated an empty sella.

Patient 2

6-year-old male (*RNU4-2*, n.64_65insT de novo)

The patient was born at 39+5 weeks of gestation (birth weight 2,888 g; length 47.1 cm; head circumference 32.0 cm), without perinatal asphyxia. Failure to thrive was noted in early infancy, with swallowing difficulties requiring tube feeding. Laryngomalacia required BiPAP support. Echocardiography and abdominal ultrasound were unremarkable. Growth failure and microcephaly persist (approximately −3 to −4 SD), along with characteristic facial features (broad nasal bridge, downslanting palpebral fissures, epicanthal folds, prominent upper vermilion, and mildly everted lips). Severe global developmental delay was documented (head control at 2 years; rolling over at 2 years 6 months; independent sitting at 3 years; no meaningful words). Ophthalmologic evaluation revealed bilateral myopic astigmatism and chorioretinal atrophy. Developmental testing showed a very low developmental quotient (e.g. K-style developmental test at 5 years: overall DQ ~11). Epileptic seizures began at around 6 years of age and are currently controlled with lacosamide and clonazepam. Brain MRI at 6 years showed mild bilateral lateral ventricular enlargement, subependymal heterotopia, and craniosynostosis.

Patient 3

19-year-old male (*RNU4-2*, n.64_65insT de novo)

Prenatal ventriculomegaly was reported. The patient was born at 38+0 weeks of gestation (birth weight 2,732 g; length 47.9 cm; head circumference 34.5 cm). Global developmental delay was documented (head control at 4 months; crawling at 3 years; independent walking at 6 years). At 19 years of age,

there were no meaningful words. Febrile seizures began at around 1 year of age, followed by afebrile seizures; antiseizure medication was initiated at 5 years. Status epilepticus episodes were reported after 8 years of age. Current seizures are described as brief events occurring approximately 1–2 times per month on perampanel and valproate.

Patient 4

26-year-old female (*RNU4-2*, n.64_65insT de novo)

Prenatal intrauterine growth restriction and ventriculomegaly were reported. The patient was born at 38 weeks of gestation (birth weight 2,350 g; length 46.0 cm; head circumference 33.6 cm). Afebrile seizures began at 6 years of age, and epilepsy was diagnosed at 9 years. The clinical course consists of refractory epilepsy, with seizure clusters occurring multiple times per month. Current antiseizure medications include levetiracetam and lacosamide. Brain MRI demonstrated reduced white matter volume and hypoplasia of the corpus callosum. Speech remained absent. Independent walking was acquired at 3 years and maintained until approximately 8 years, followed by gradual decline; the patient currently ambulates with assistance (hand-held support). No history of fractures was reported.

Patient 5

9-year-old female (*RNU4-2*, n.64_65insT de novo)

Prenatal intrauterine growth restriction and microcephaly were reported. The patient was born preterm at 36 weeks of gestation via spontaneous vertex delivery with a birth weight of 2.4 kg. Postnatally, she developed global developmental delay with feeding difficulties and failure to thrive. Generalized hypotonia and short stature were also noted. She has been treated for bilateral inguinal hernia, bilateral developmental dysplasia of the hips, and autism spectrum disorder. Dysmorphic features included large prominent ears, myopathic facies, deep-set eyes, a tented upper lip with an everted lower lip, a protruding tongue, and a short philtrum with full lips. Other system examinations were reportedly unremarkable. She attends a special needs school and is dependent on others for self-care and activities of daily living. Neuroimaging showed cerebral and cerebellar atrophy.

Patient 6

16-year-old female (*RNU4-2*, n.64_65insT de novo)

The patient was born at 39+6 weeks of gestation (birth weight 2,632 g). Nystagmus was noted in the neonatal period. Failure to thrive (height/weight/head circumference all below −2 SD) and hypotonia were noted at 4 months of age. Developmental milestones included head control at 6 months, sitting at 1 year, and standing with support (with orthoses) at 4 years 11 months; the patient can ambulate using a walker. No clearly meaningful words were reported. At 1 year 11 months, prolonged focal status epilepticus (left-predominant) occurred, and the patient was diagnosed with hemiconvulsion–

hemiplegia–epilepsy (HHE) syndrome with residual incomplete hemiparesis (left upper and lower limbs). Seizures have been absent since age 5 years 8 months. The patient was treated with valproate; at 12 years, acute pancreatitis suspected to be valproate-related prompted a switch to lacosamide, which is currently being tapered.

Patient 7

8-year-old male (*RNU4-2*, n.64_65insT de novo)

Mild fetal growth restriction was reported prenatally. Growth and developmental delay were recognized at an 8-month health check. Work-up identified Chiari malformation type I, treated with foramen magnum decompression and C1 laminectomy at 1 year 2 months of age. At 1 year 9 months, moderate developmental delay and growth impairment were documented (poor weight gain ~−2.8 SD, short stature ~−2.6 SD, microcephaly ~−2.2 SD). At 8 years old, the patient had acquired ambulation and a few simple words of speech. Developmental delay and estimated intellectual disability are currently severe, and epilepsy has developed. Additional findings included mild microcytic hypochromic anemia unresponsive to iron supplementation and mildly elevated serum copper.

Patient 8

11-year-old male (*RNU4-2*, n.64_65insT de novo)

Prenatal intrauterine growth restriction and bilateral ventriculomegaly were reported. The patient was born at 37+1 weeks of gestation by elective Caesarean delivery for breech presentation (birth weight 2,205 g; length 45 cm; head circumference 31 cm). Neonatal care included nasal CPAP and tube feeding; discharge occurred at approximately day 45. At 11 years 3 months, height was 136 cm (−1.06 SD), weight 21.8 kg (−1.86 SD), and head circumference 47.5 cm (approximately −4 SD).

Developmental delay and microcephaly were noted (head control at 9 months; rolling over at 10 months; sitting at 14 months; walking with orthopedic devices at 8 years). There is no history of epilepsy; however, recurrent febrile seizures have occurred with nearly every febrile episode. Behavioral concerns, including self-injurious behavior (e.g., head banging and hair pulling), were reported from around 4 years of age. Brain MRI demonstrated ventriculomegaly and hypoplasia of the corpus callosum.

Patient 9

19-year-old female (*RNU4-2*, n.64_65insT de novo)

The patient was born at 38 weeks of gestation by vaginal delivery (birth weight 2,652 g; length 45.4 cm; head circumference 31.0 cm). Feeding difficulties were noted from birth. At 3 months, microcephaly, micrognathia, hypotonia, and failure to thrive were documented. Developmental milestones included head control at 5 months, independent sitting at 1 year, and walking at 3 years.

She spoke several words at 19 years of age. Developmental testing at 14 months showed a low developmental quotient (Enjoji developmental test: overall DQ 41). She had stereotypic hand movements and a wide-based gait. Seizure-like episodes began at around 3 years of age (vomiting with perioral cyanosis and upward eye deviation), with recurrent events and epileptiform discharges on EEG; valproate was initiated and subsequently controlled seizures. Later EEGs reportedly showed no epileptiform activity. Brain MRI showed diffuse ventriculomegaly, bilateral frontal/temporal atrophy, and hypoplasia of the corpus callosum. Additional features include scoliosis, chronic constipation, recurrent respiratory and gastrointestinal infections, and primary hypothyroidism.

Patient 10

13-year-old female (*RNU4-2*, n.65A>G de novo)

The patient was born at 37+3 weeks of gestation (birth weight 2,810 g). Hypotonia was recognized at 6 months of age. Generalized joint and skin laxity were noted in early childhood. Motor delay was present, with independent walking at 2 years 4 months. Speech and language delay were noted (two-word sentences at 4 years 10 months). At 5 years, gait remained unsteady and stair climbing required handrail support. At 13 years, the patient attends a special needs school (junior high school) with intellectual disability (IQ 46). Joint laxity has become less prominent over time.

Patient 11

5-year-old female (*RNU4-2*, n.68A>G de novo)

Prenatal fetal growth restriction and ventriculomegaly were noted from 25 weeks of gestation. The patient was born at 37+6 weeks by emergency Caesarean section for non-reassuring fetal status (birth weight 1,864 g). Developmental delay was profound: at 5 years of age, head control has been achieved, but rolling over and independent sitting are not possible; there are no meaningful words. Seizures began at 2 months (left-sided tonic–clonic), prompting initiation of antiseizure therapy. Laryngomalacia and renal hypoplasia were also reported. Epilepsy is currently controlled with valproate and perampanel.

Patient 12

61-year-old male (*RNU4-2*, n.18_19insA; familial inheritance suspected)

The patient presented with progressive visual impairment and a family history notable for multiple relatives, including his father, a paternal sibling, and a niece, who had similar visual symptoms. He also had a medical history of Brugada syndrome. An abnormality was first detected on fundus examination during a routine health checkup at 40 years of age, although he had minimal subjective symptoms at that time. From his 40s onward, he increasingly bumped into objects while walking. At 56 years of age, he experienced a rapid worsening of visual acuity, photophobia, and progressive

constriction of the visual field, prompting referral. On initial examination, best-corrected visual acuity was 20/25 in the right eye (OD) with a refraction of −1.00 D sphere and 20/16 in the left eye (OS) with a refraction of −1.00 D cylinder at 165 degrees. Intraocular pressure was within normal limits in both eyes, and anterior segment examination revealed a moderate cataract in the right eye. Goldmann perimetry demonstrated symmetrical, severe bilateral visual field constriction with a residual central island of approximately 10 degrees. Optical coherence tomography showed parafoveal outer retinal atrophy with defects of the ellipsoid zone, indicating structural abnormalities of the photoreceptor layer. Full-field electroretinography revealed an absent rod response and markedly reduced cone responses, consistent with retinitis pigmentosa. The patient subsequently underwent cataract surgery in the right eye, after which best-corrected visual acuity improved to 20/16 OD with a refraction of +0.25 D sphere / −1.00 D cylinder at 95° and 20/22 OS with a refraction of +0.25 D sphere / −1.00 D cylinder at 170°. Although progression of the cataract in the left eye was observed during follow-up, there was no evident progression of retinal degeneration.

Patient 13

9-year-old male (*RNU5B-1*, n.42_43insA de novo)

The patient was born at 37+3 weeks of gestation (birth weight 2,450 g) without perinatal asphyxia. Neonatal seizures occurred shortly after birth and were treated with phenobarbital until 5 months of age. At 9 months, the patient was hospitalized for recurrent apnea and hypoxemia; EEG supported epileptic apnea, and antiseizure therapy was switched to clobazam, which improved the status epilepticus and long apnea episodes. The subsequent course included ongoing seizures, growth problems, global developmental delay, feeding difficulties with sialorrhea, and hypotonia/weakness. Additional findings, such as relative macrocephaly, pectus excavatum and spinal/cord anomalies (lipomyelomeningocele with tethered cord), were also reported. The patient is able to walk unsupported with gait ataxia but has acquired no meaningful words. Dysmorphic facial features included hypotonia, long palpebral fissures, downslanted palpebral fissures, sparse eyebrows, broad nasal bridge, tented upper lip, everted lower lip, low-set ears. Brain MRI reportedly demonstrated frontotemporal atrophy and hypoplasia of the corpus callosum.

Patient 14

14-year-old female (*RNU2-2*, n.4G>A de novo)

The patient was born at 40 weeks of gestation by Caesarean section for breech presentation (birth weight 2,490 g (-1.2 SD); length 48.0 cm (-0.8 SD); head circumference 33.5 cm (+0.2 SD)), without perinatal asphyxia. Decreased fetal movements were noted prenatally. Growth tracked at approximately −1 SD during childhood; however, at 14 years of age, her height was 145.0 cm (−2.1 SD), weight 30 kg (−3.0 SD), and head circumference 52.5 cm (−1.2 SD), indicating short stature and

relative macrocephaly. Physical examination showed thick lips and thick eyebrows, without other notable dysmorphic features. Developmental delays were apparent from infancy with delayed motor milestones (head control at 3 months, independent sitting at 11 months, pulling to stand at 13 months, cruising at 15 months, and independent walking at 24 months). She developed no meaningful spoken words, did not respond to her name, and showed limited interest in people or her surroundings, leading to a diagnosis of autism spectrum disorder. Psychological assessment at school age demonstrated severe developmental delay (overall DQ = 20), without evidence of developmental regression. At 14 years of age, she walked independently and demonstrated functional comprehension in everyday situations but had no meaningful spoken words; communication was primarily nonverbal, with limited use of sign language. She was able to feed herself with a spoon and was independent in dressing and undressing. She required diapers and was able to report after toileting. At 1 year 10 months of age, she developed left-predominant generalized tonic–clonic seizures that progressed to status epilepticus. Thereafter, seizures recurred irrespective of fever. Seizure control was achieved with antiseizure medications, including carbamazepine, levetiracetam, and valproate. At 14 years of age, EEG findings were consistent with Lennox–Gastaut syndrome. Brain MRI at 14 years of age showed no significant abnormalities.

Patient 15

7-year-old female (*RNU2-2*, n.58T>G (paternal) and n.66A>C (maternal) compound heterozygote)

The patient is a 7-year-old girl with epilepsy, global developmental delay, abnormal/involuntary movements, and poor weight gain. Developmental delay and failure to thrive were recognized at around 1 year of age. Motor development was relatively preserved until head control was achieved, but sitting was delayed and walking remained difficult, requiring gait training. Additional features recorded in the notes include microcephaly, absent speech, autistic-like traits, and a happy affect; facial features included a broad nasal bridge. Seizures became difficult to control after 3 years of age; multiple antiseizure medications (5–6 agents) were trialed with limited efficacy.

Patient 16 and 17

13-year-old female and 6-year-old female (*RNU2-2*, n.100T>G (paternal) and n.155C>G (maternal) compound heterozygote)

The patient is a 13-year-old girl with a history of global developmental delay since infancy. She showed delayed developmental milestones, achieving army crawling at 12 months of age. She developed epilepsy at 21 months, and electroencephalography revealed epileptiform abnormalities. Independent ambulation was achieved at 3 years and 8 months; however, her seizures subsequently worsened, and her motor function regressed. At present, she is only barely able to maintain a sitting

position. She has no meaningful spoken language and is fed via gastrostomy. She has a severe intellectual disability and experiences recurrent generalized tonic seizures. Her younger sister is currently 6 years old. She is able to crawl on all fours but has no meaningful speech and also has severe intellectual disability. Although epileptic seizures ares suspected, they have not yet been clearly confirmed. She repeatedly exhibits Rett syndrome–like hand-wringing movements and bruxism. Both siblings have a height and weight above the standard range, and a body size that is at or above the average for their age. Her older sister has a head circumference of 50 cm (−2.3 SD), consistent with microcephaly, whereas the younger sister's head circumference is within the normal range. Both have chronic constipation.

Patient 18

13-year-old male (*RNU4ATAC*, n.13C>T and n.13C>G compound heterozygote)

Prenatally, hydrocephalus and fetal growth restriction were suspected. The patient was born at 38+6 weeks of gestation (birth weight 1,796 g) without asphyxia; neonatal hypoglycemia required intravenous therapy. The patient was discharged on day 22. The medical course includes recurrent hospitalizations for respiratory infections/asthma. At 4 years 10 months, motor paralysis of both lower extremities developed, and the patient was treated for suspected Guillain–Barré syndrome or myelitis. Motor function subsequently recovered to the pre-morbid baseline. The clinical phenotype includes severe short stature (approximately −4 SD), global developmental delay, and a Dandy–Walker variant (enlarged cisterna magna with cerebellar hypoplasia). Additional multisystem findings included urethral/urogenital anomalies, brachydactyly, atopic dermatitis, and difficult-to-manage type 1 diabetes.

Patient 19

4-year-old male (*RNU4ATAC*, n.13C>T (maternal) and n.119T>C (paternal) compound heterozygote)

The patient was born at 35 weeks of gestation by emergency Caesarean section. Birth weight was 1,419 g, small for gestational age. The patient has atopic dermatitis, recurrent infections, relative macrocephaly, scrotal hypoplasia and bilateral retractile testes. Growth impairment has persisted: at the latest evaluation, height was 92.1 cm (approximately −3 SD), weight 13.8 kg (approximately −1.5 SD), and head circumference 48.0 cm (approximately −1.5 SD). Developmental delay has been described as mild-to-moderate (approximately 6–12 months behind peers). At approximately 3 years 11 months, the patient produced three-word sentences and was attending daycare with developmental support. At 4 years 8 months, the patient developed acute motor paralysis and was treated for Guillain–Barré syndrome. Brain MRI showed ventriculomegaly and mild aqueductal stenosis. Ophthalmologic findings included enlarged optic disc cupping and suspected optic atrophy. The patient was also

clinically diagnosed as having Russell–Silver syndrome. Recombinant growth hormone therapy has been started for SGA-related short stature, with improved growth velocity.

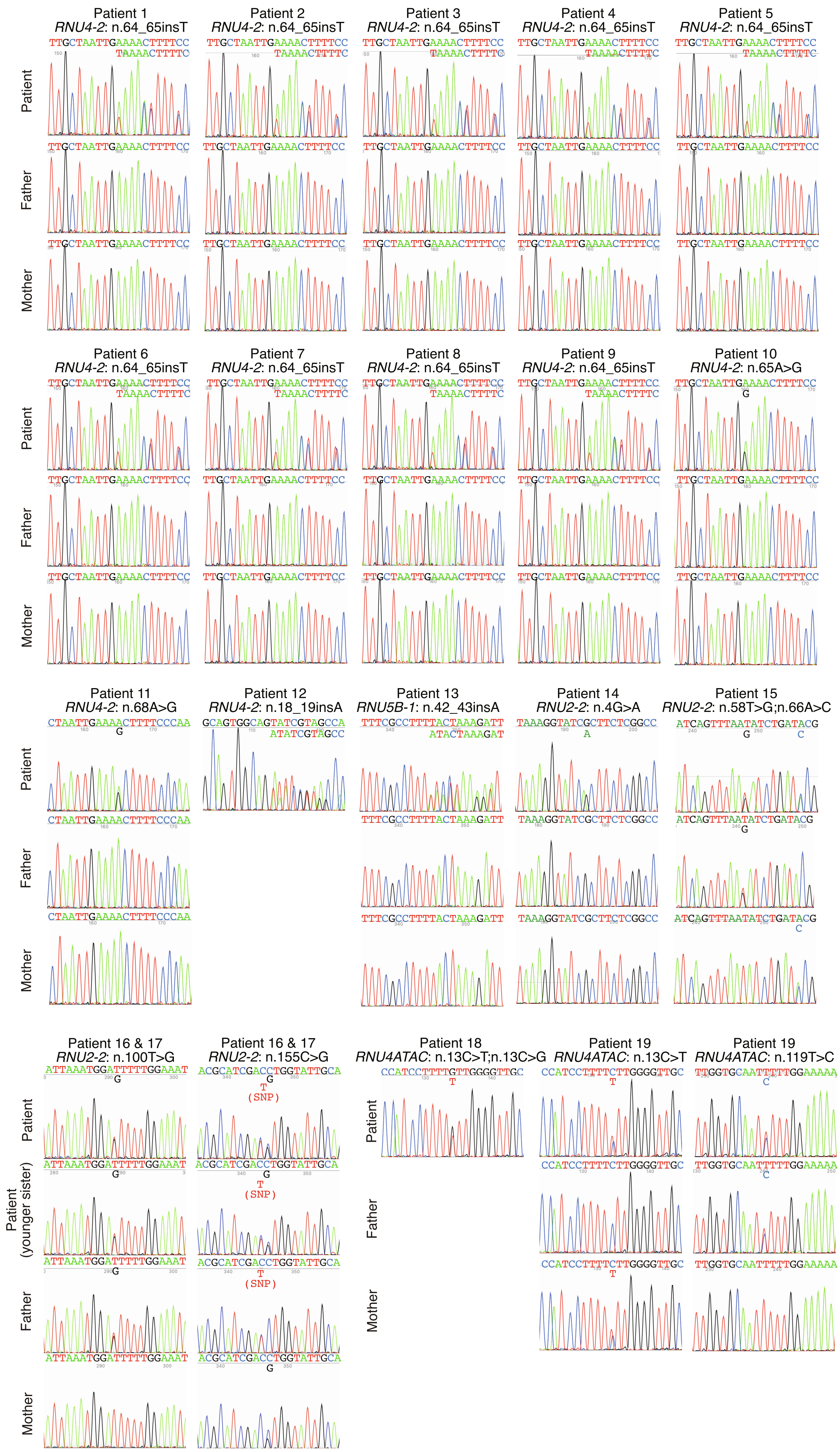

Figure S1. Sanger sequencing validation of identified variants

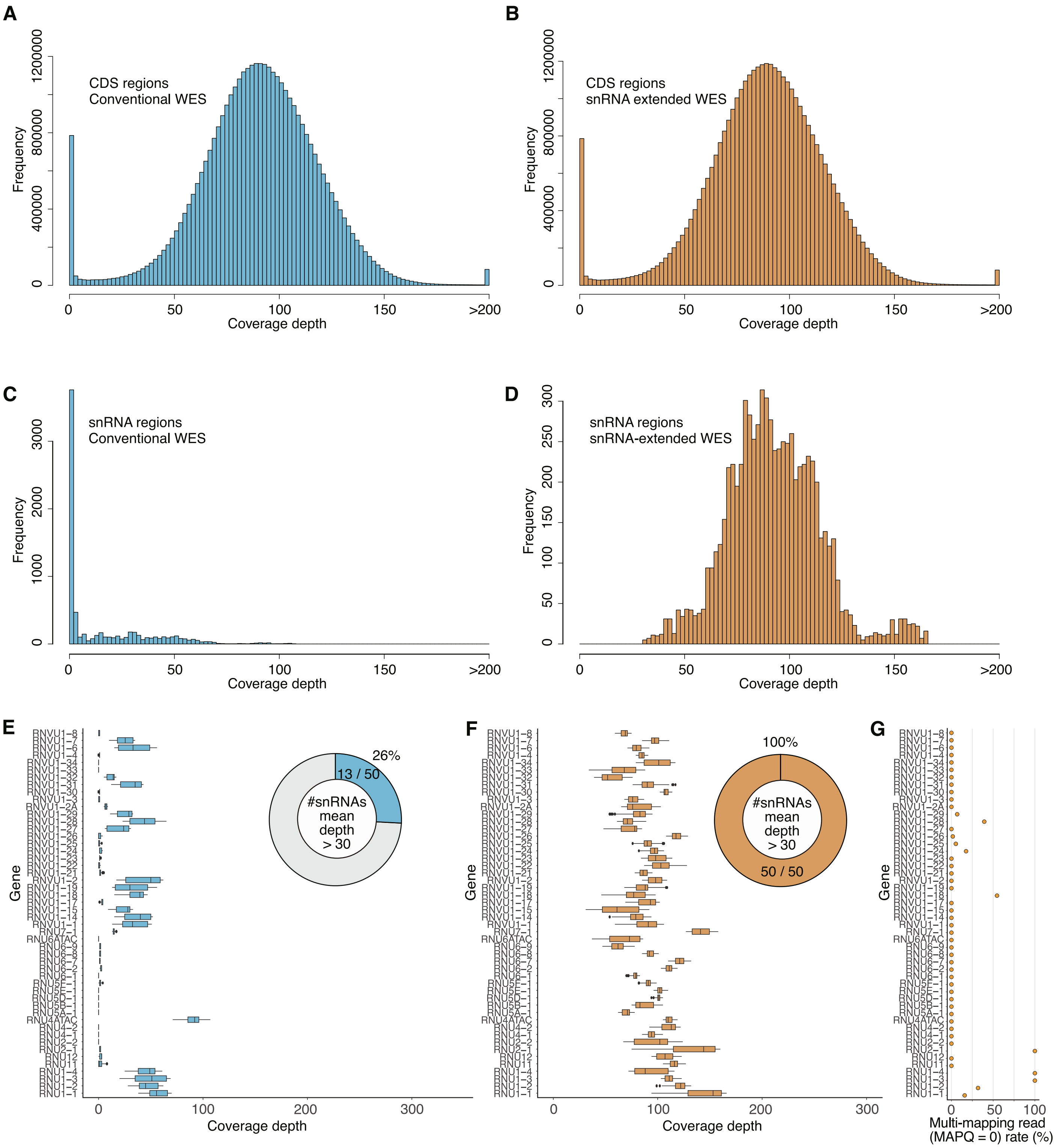

Figure S2. Coverage comparison between conventional WES and snRNA-extended WES for HG001

(A, B) Histograms showing coverage depth across all CDS regions obtained by conventional WES (A) and snRNA-extended WES (B). Data shown in panels (A) through (F) were generated from the HG001 sample using either conventional WES or snRNA-extended WES, with sequencing output normalized to 9 Gb. All samples were run on the same platform.

(C, D) Histograms showing coverage depth across all 50 targeted snRNA regions obtained by conventional WES (C) and snRNA-extended WES (D).

(E, F) Box plots showing coverage depth for each of the 50 snRNA genes obtained by conventional WES (E) and snRNA-extended WES (F). Center line, median; box limits, upper and lower quartiles; whiskers, 1.5× interquartile range (IQR); dots, values beyond 1.5× IQR. Pie charts in the upper right indicate the proportion of snRNA genes with a mean coverage depth greater than 30.

(G) Multi-mapping read (MAPQ = 0) fraction for each snRNA gene.

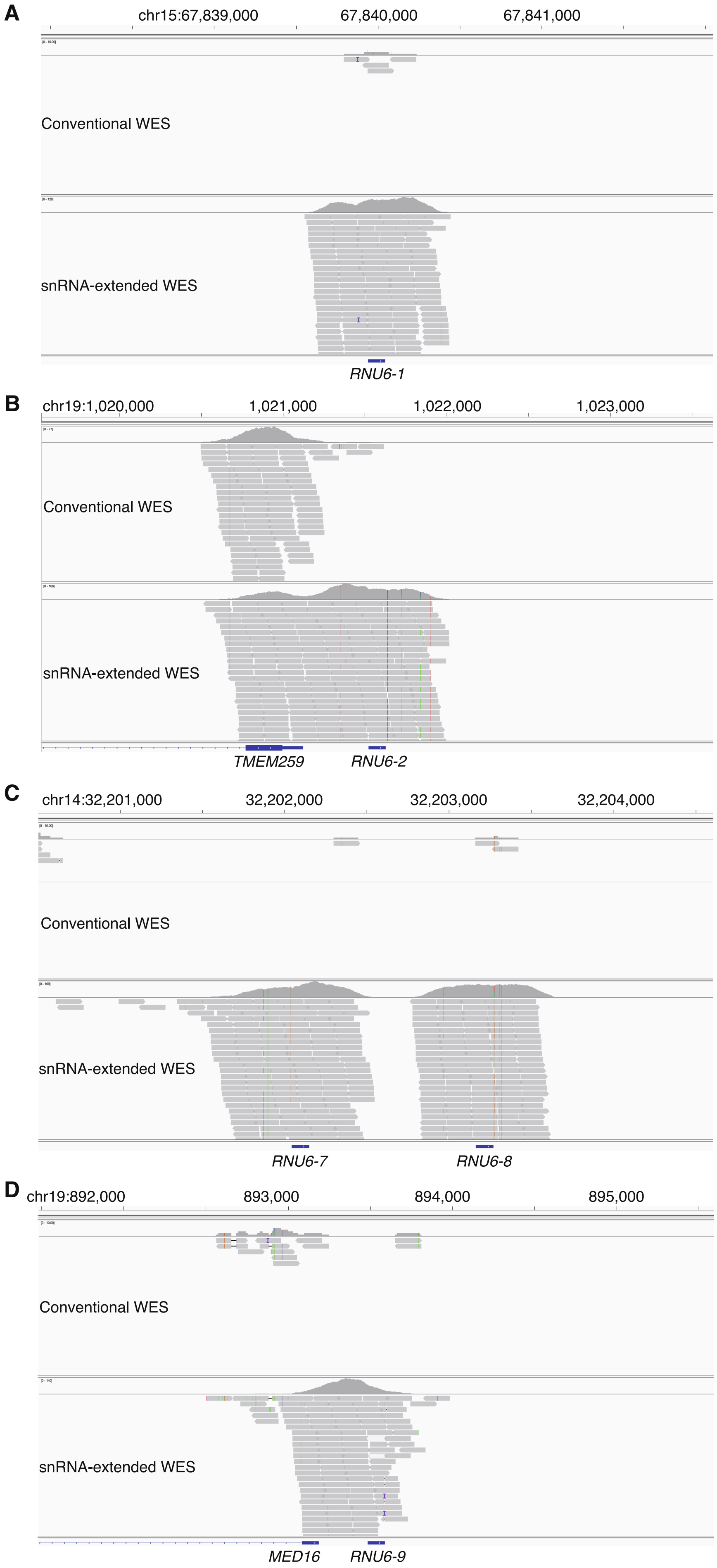

Figure S3. Coverage of *RNU6* genes

(A - D) IGV images comparing conventional WES and snRNA-extended WES performed on genomic DNA from HG002 sample at (A) *RNU6-1*, (B) *RNU6-2*, (C) *RNU6-7* and *RNU6-8*, and (D) *RNU6-9*.
